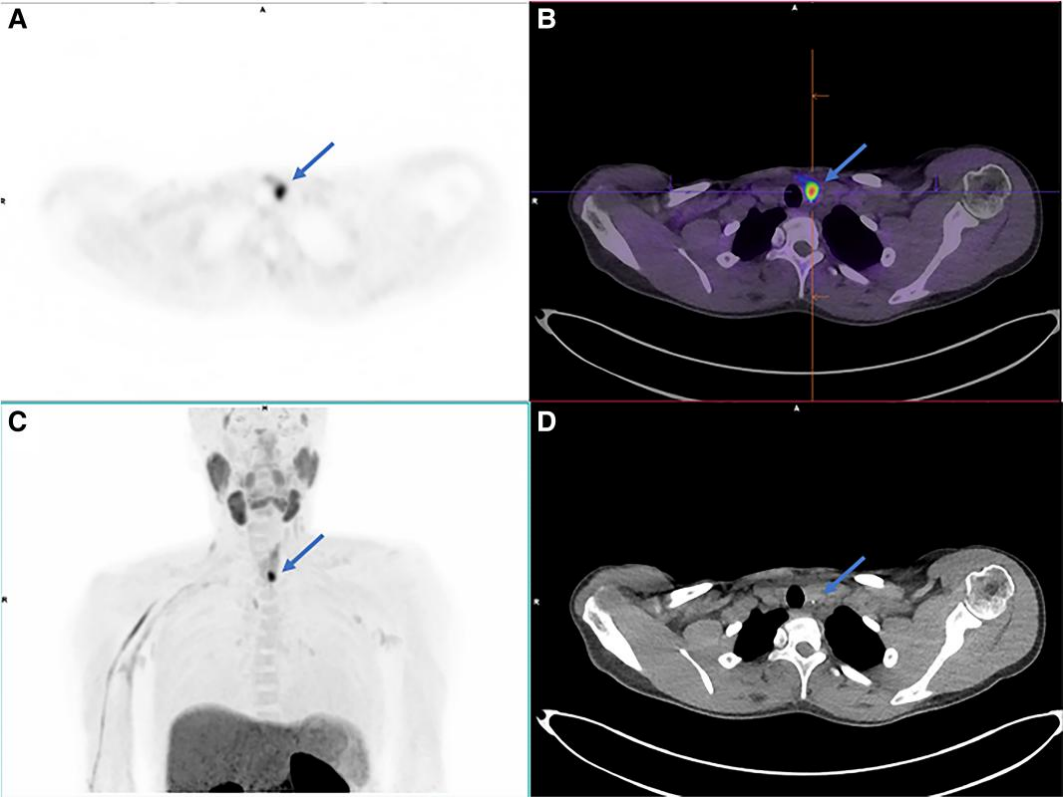# Successful Localization of Recurrent MEN-1-Associated Hyperparathyroidism With 18F-Fluorocholine PET/CT

**DOI:** 10.1210/jcemcr/luae222

**Published:** 2024-12-04

**Authors:** Elizabeth Wootton, Clement Wong, Amanda Love, David A Pattison

**Affiliations:** Department of Endocrinology and Diabetes, Royal Brisbane and Women's Hospital, Brisbane, QLD, 4006, Australia; Faculty of Medicine, University of Queensland, Brisbane, QLD, 4006, Australia; Faculty of Medicine, University of Queensland, Brisbane, QLD, 4006, Australia; Department of Surgery, Royal Brisbane and Women's Hospital, Brisbane, QLD, 4006, Australia; Department of Endocrinology and Diabetes, Royal Brisbane and Women's Hospital, Brisbane, QLD, 4006, Australia; Faculty of Medicine, University of Queensland, Brisbane, QLD, 4006, Australia; Faculty of Medicine, University of Queensland, Brisbane, QLD, 4006, Australia; Department of Nuclear Medicine, Royal Brisbane and Women's Hospital, Brisbane, QLD, 4006, Australia

**Keywords:** hyperparathyroidism, MEN-1, fluorocholine

## Image Legend

A 29-year-old woman with multiple endocrine neoplasia type 1 (MEN-1) and Turner syndrome was evaluated for recurrent primary hyperparathyroidism. In 2014, she underwent a 2.5-gland parathyroidectomy and thymectomy with removal of the left superior, left inferior (partially intrathyroidal tissue left in situ), and right inferior glands. The right superior gland was not located. She maintained eucalcemia until 2018. On recent testing, her serum albumin-adjusted calcium measured 2.7 mmol/L (reference range, 2.10-2.65 mmol/L; 10.8 mg/dL [8.4-10.6 mg/dL]), ionized calcium 1.47 mmol/L (1.16-1.32 mmol/L; 5.9 mg/dL [4.65-5.29 mg/dL]) with parathyroid hormone concentration 14 pmol/L (1.0-7.0 pmol/L; 132 ng/L [9.4-66 ng/L]). She remained asymptomatic without nephrocalcinosis but significant decline in bone mineral density was evident on serial dual-energy x-ray absorptiometry.

Parathyroid localization studies with sestamibi and neck ultrasound were equivocal for 2 parathyroid adenomas at the left hemithyroid. Four-dimensional computed tomography (4DCT) was unremarkable. ^18^F-fluorocholine positron emission tomography/computed tomography (PET/CT) clearly identified hyperfunctioning parathyroid tissue at the lower pole of the left hemithyroid. She proceeded to parathyroidectomy with histology confirming parathyroid adenoma and subsequent postoperative biochemical cure.

^18^F-fluorocholine PET/CT shows higher accuracy with lower radiation exposure than existing imaging techniques for localizing hyperfunctioning parathyroid tissue [1]. It is particularly valuable in the MEN-1 population, who frequently have multiglandular parathyroid disease which may be ectopic in location [2].

The image shows ^18^F-fluorocholine PET/CT: A, axial PET; B, fused PET/CT; C, PET maximum intensity projection; and D, CT clearly identifying hyperfunctioning parathyroid tissue at the lower pole of the left hemithyroid (blue arrow).

**Figure:**